# QSurface: fast identification of surface expression markers in cancers

**DOI:** 10.1186/s12918-018-0541-6

**Published:** 2018-03-19

**Authors:** Yourae Hong, Choa Park, Nayoung Kim, Juyeon Cho, Sung Ung Moon, Jongmin Kim, Euna Jeong, Sukjoon Yoon

**Affiliations:** 1 Center for Advanced Bioinformatics & Systems medicine, Department of Biological Sciences, Sookmyung Women’s University, Hyochangwon-gil 52, Yongsan-gu, Seoul, 140-742 Republic of Korea; 2 Department of Biological Sciences, Sookmyung Women’s University, Seoul, 140-742 Republic of Korea

**Keywords:** Cancer transcriptome, Cancer mutations, Antibody-drug conjugates, Software development

## Abstract

**Background:**

Cell surface proteins have provided useful targets and biomarkers for advanced cancer therapies. The recent clinical success of antibody-drug conjugates (ADCs) highlights the importance of finding selective surface antigens for given cancer subtypes. We thus attempted to develop stand-alone software for the analysis of the cell surface transcriptome of patient cancer samples and to prioritize lineage- and/or mutation-specific over-expression markers in cancer cells.

**Results:**

A total of 519 genes were selected as surface proteins, and their expression was profiled in 14 cancer subtypes using patient sample transcriptome data. Lineage/mutation-oriented analysis was used to identify subtype-specific surface markers with statistical confidence. Experimental validation confirmed the unique over-expression of predicted surface markers (MUC4, MSLN, and SLC7A11) in lung cancer cells at the protein level. The differential cell surface gene expression of cell lines may differ from that of tissue samples due to the absence of the tumor microenvironment.

**Conclusions:**

In the present study, advanced 3D models of lung cell lines successfully reproduced the predicted patterns, demonstrating the physiological relevance of cell line-based 3D models in validating surface markers from patient tumor data. Also QSurface software is freely available at http://compbio.sookmyung.ac.kr/~qsurface.

**Electronic supplementary material:**

The online version of this article (10.1186/s12918-018-0541-6) contains supplementary material, which is available to authorized users.

## Background

Cell surface proteins have provided major targets and biomarkers for anticancer therapies. In colorectal cancer, the expression of surface proteins such as CDH17, CD138 and members of the integrin family is related with tumor progression [1]. Another surface protein, SEZ6L2 was identified a novel prognostic marker in non-small cell lung cancer (NSCLC) [2]. Epidermal growth factor receptor (EGFR) is over-expressed cell types and plays a key role in cancer progression. Indeed, many drugs targeting EGFR have been developed [3]. In addition, HER2, a plasma membrane-bound protein and member of the ErbB family, is significantly over-expressed in 10–15% of breast cancers, referred to HER2-positive breast cancer [4, 5].

More recently, cell surface proteins have been successfully used as targets for antibody-drug conjugates (ADC) as part of cancer therapy [6–8]. ADCs are composed of antibodies for targeting and cytotoxic drugs and linker proteins for attaching to and cleaving the target. Once ADCs reach and attach to the target antigen on the cancer cell surface, receptor-mediated endocytosis internalizes the antibody and cytotoxic drug. Thus, surface antigens for ADCs should exhibit tumor-specific expression for the selective targeting of ADCs, and facilitate receptor-mediated endocytosis. A complete list of tumor-specific cell surface markers will help identify potential antigens for this type of advanced therapy.

The Cancer Genome Atlas (TCGA) is one of the largest datasets from pan-cancer analyses [9]. The released multi-omics dataset includes genome, transcriptome and proteome data for tissue samples from thousands of cancer patients, covering ~ 30 cancer types. The proteome dataset, generated using reverse-phase protein arrays (RPPA), is limited in the availability of specific antibodies [10]. For TCGA, the expression data for a few hundred proteins are available [11]. However, TCGA transcriptome data includes the expression profiles of ~ 20,000 genes, enabling the identification of selectively overexpressed genes corresponding to surface proteins [12].

As an analysis tool to find differentially expressed genes, cBioPortal [13, 14] is useful but has limitation of comparative analyses using two or more omics datasets. For example, cBioPortal doesn’t provide extensive analyses using both somatic mutation and gene expression datasets. In addition, cell surface genes and proteins are not classified in cBioPortal.

In the present study, we developed stand-alone software, QSurface, to analyze lineage- and/or mutation-specific cell surface transcriptome marker from cancer patients’ samples obtained from TCGA. Selected expression markers were validated at the protein level using lung adenocarcinoma (LUAD) cell lines. Notably, the gene expression of extracellular/membrane proteins exhibits inconsistent patterns between cell lines and patient tissue samples [15]. In the present study, we attempted to overcome this problem using advanced 3D sphere-based assays which provided a physiologically relevant microenvironment for the tested cell lines [16]. The present software and assay method will provide fast and efficient tools to identify novel tumor-specific cell surface markers for advanced cancer therapies such as ADCs.

## Method

### Data acquisition

RNA sequencing version 2 (RNASeqV2) data from patients’ tumor and normal tissue samples were downloaded from TCGA website (http://cancergenome.nih.gov/) in 2015. The RNASeqV2 data were sequenced using the Illumina HiSeq 2000 and Illumina Genome Analyzer (GA) platforms. We selected 658 tumor samples with matched normal samples obtained from the same patient in 14 cancer types, satisfying the requirement more than 10 samples (Additional file 1: Table S1). The expression level of each gene was normalized using RNA-Seq by Expectation Maximization (RSEM) count estimates method and we converted the data to the log2 scale. For breast invasive carcinoma (BRCA), two tumor patients were duplicated using primary and metastatic samples. We excluded two metastasis samples.

TCGA provides multi-dimensional datasets, which means that one samples has genotype and expression data together. We obtained the somatic mutation dataset from cBioPortal. The curated dataset has been processed from published literature. Somatic mutations are curated and annotated with information of variant effects, predicted from SIFT [17] and Polyphen-2 [18] algorithms. To analyze only non-synonymous mutations including truncating mutation and deleterious missense mutation, we excluded neutral mutations predicted from two algorithms. The criteria of non-synonymous mutations are SIFT score < 0.05 or Polyphen-2 score > 0.85. Gene expression data were integrated with these processed mutation data. After annotating tumor samples, 555 tumor samples are remained for analysis (Additional file 1: Table S1).

### Selection of cell surface genes

We selected ‘cell surface’ (Gene Ontology term GO:0009986) from the cellular components category to identify cell surface genes from the AmiGO website, October 2016 [19, 20]. A total of 524 genes belonging to the ‘cell surface’ category were located in the external part of the cell wall or plasma membrane. Among of these genes, 519 genes remained for analysis after mapping using the TCGA RNASeqV2 data.

### Statistical analysis

To identify differentially expressed cell surface genes, we used log2 delta and t-test *P*-values. We calculated log2 delta as the average difference in the expression levels for lineage-specific cell surface genes between tumor and normal samples and for mutation-specific cell surface genes between mutant and wild-type tumor samples per lineage.

#### 2D cell culture

Three types of cell lines, an STK11 mutant type (A549, H460, H23, and H1993), STK11 wild type (H522, H322M, HCC-827, and H1975), and STK11-recoverd type (A549-STK11, H460-STK11, H23-STK11, and H1993-STK11) were used for validation experiments. HCC-827 and H1975 cells were obtained from the American Type Culture Collection (ATCC, Manassas, VA, USA), respectively. All other STK11 mutant and wild-type cell lines were obtained from the National Institutes of Health, National Cancer Institute (NCI, Frederick, MD, USA). STK11 mutant cell lines and wild-type cell lines were cultured in RPMI 1640 (HyClone Laboratories, Logan) supplemented with 10% fetal bovine serum (HyClone Laboratories) and 1% antibiotics (GIBCO BRL, Thermo Fisher Scientific). STK11-recoverd cell lines were cultured in the same medium with added 1μg/ml puromycine. A total of 1~ 3 × 10^5^ cells per well were seeding on a 6well culture plate for monolayer cell culture during 5 days.

#### 3D cell culture

Cancer stem-like cell (CSLC) spheres were cultured in serum-free conditioned DMEM/F-12 medium supplemented with 20-ng/ml EGF, 20-ng/ml basic fibroblast growth factor, and B27 (Thermo Fisher Scientific). The cells were maintained in a humidified atmosphere of 5% CO_2_ and 95% air at 37 °C and the culture medium was refreshed every 2 to 3 days. The culture plates for stem-like cells (SLCs) were coated with a 5-mg/ml solution of poly-2-hydroxyethyl methacrylate (Sigma-Aldrich) in 95% ethanol. The same amount of cells as 2D cell culture was seeded in a 6-well plate for sphere culture.

### Western blot

Total cell extracts were prepared by incubating the cells in lysis buffer (RIPA Cell lysis buffer containing 150 mM sodium chloride, 1% Triton X-100, 1% sodium deoxycholate, 0.1% SDS, 50 mM Tris-HCl, pH 7.5, and 2 mM EDTA, sterile solution, GenDEPOT) on ice for 30 min. Cell debris was removed by centrifugation, and the total protein levels in the supernatants were quantified using the Bradford method (Bio-Rad). Equal amounts of protein (50 μg) were heated at 95 °C for 5 min, electrophoretically resolved using 12% SDS-PAGE, and then transferred to nitrocellulose membranes (Millipore). The membranes were blocked with TBST [20 mM Tris-HCl, pH 7.6, and 0.1% Tween-20] containing 5% skim milk for 1 h and then hybridized as indicated to specific primary antibodies (1:1000 dilution) at 4 °C overnight. The membranes were washed and hybridized to HRP-conjugated secondary antibodies for 1 h at room temperature. Specific bands were visualized using an enhanced chemiluminescence (ECL) detection system (Thermo, Logan, UT, USA) and an LA3000 luminescence image analyzer (Fujifilm, Tokyo, Japan). Antibodies against MUC4 and SLC7A11 were purchased from Abcam PLC. Antibodies against Mesothelin and GAPDH were purchased from Cell Signaling. GAPDH was used as a loading control. The anti-STK11 antibody was purchased from Santa Cruz.

## Results and discussion

### Implementation of QSurface

**Fig. 1:**
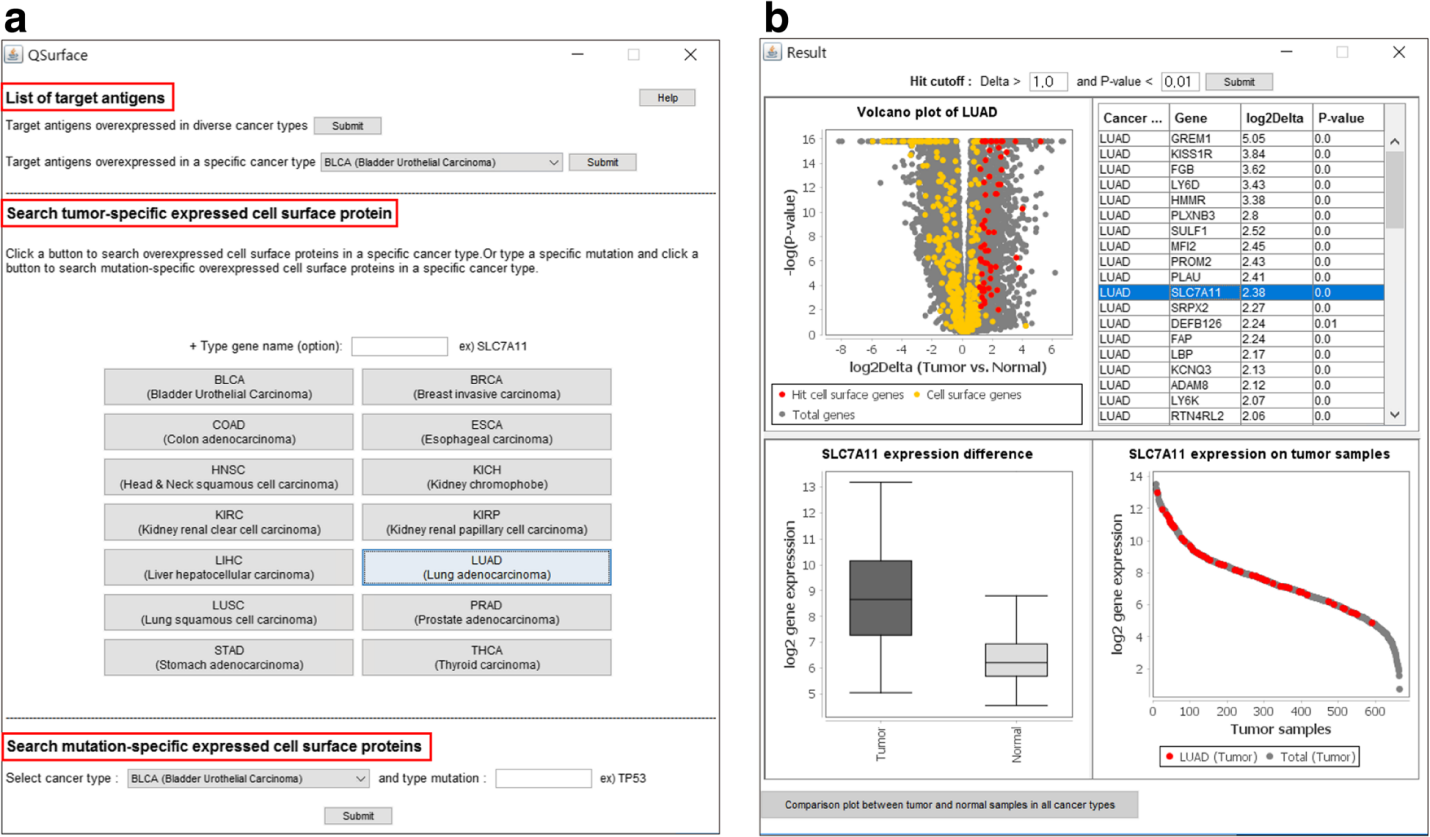
Overview of QSurface. **a** Graphical user interface of QSurface front page. **b** Snapshot of lineage-oriented profiling for lung adenocarcinoma (LUAD)

We implemented QSurface, a tool for exploring lineage- and/or mutation-specific gene expression of all potential surface proteins (Fig. 1a). Genes for surface proteins were defined using the category information in Gene Ontology database [20].

Users can browse differential gene expression of 29 known target antigens that are currently under phase I or II clinical trials [6, 21, 22] (Additional file 1: Table S2). Candidate genes are separated into two groups, overexpressed in diverse tumors and over-expressed in specific tumors.

To identify lineage-specific gene expression, users can search differentially expressed genes by selecting a specific lineage, for example, lung adenocarcinoma tumor LUAD (Fig. 1a). A volcano plot is used to display the expression differences between tumor and normal samples. Firstly, a total of 20,531 genes and 519 cell surface genes are visualized in grey and yellow colors, respectively. The significant differentially expressed genes are shown in red color in the plot and listed as a table after submitting criteria (log2Delta > 1.0 and *P*-value < 0.01) in the top of the window (Fig. 1b upper). By clicking a hit gene (SLC7A11) from the list, the box plot and the waterfall plot will be popped up to show the expression pattern between tumor and normal samples and the lineage specificity (in this case LUAD) among all samples for the selected hit gene SLC7A11 (Fig. 1b bottom). Lastly, the comparison of gene expression pattern between tumor and normal samples among all cancer types is available for the hit gene by clicking “Comparison plot” in the bottom of the window.

**Fig. 2:**
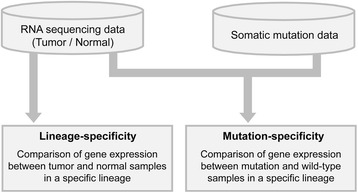
Dataflow and data processing of QSurface. Lineage-oriented profiling uses paired tumor and normal samples in RNA sequencing data, and mutation-oriented profiling uses somatic mutation data and only tumor samples in RNA sequencing data

Furthermore, mutation-specific hits can be also displayed by adding mutation criteria together with lineage information. Users can obtain over-expressed hits enriched in mutant samples over wild type samples for a given lineage. The overall data processing and analytical flow are described in Fig. 2.

To summarize, QSurface is a tool to analyze lineage- and/or mutation-specific gene expression of all potential surface proteins between tumor and normal samples or mutant and wild-type samples. It is helpful to find potential cell surface marker using difference of gene expression with statistical confidence. QSurface is a stand-alone Java tool that can be run on any operating system. JavaStat and JFreeChart library packages were used to calculate Student’s t-test and draw all plots, respectively.

### Lineage-based analysis of cell surface genes and known target antigens

**Fig. 3:**
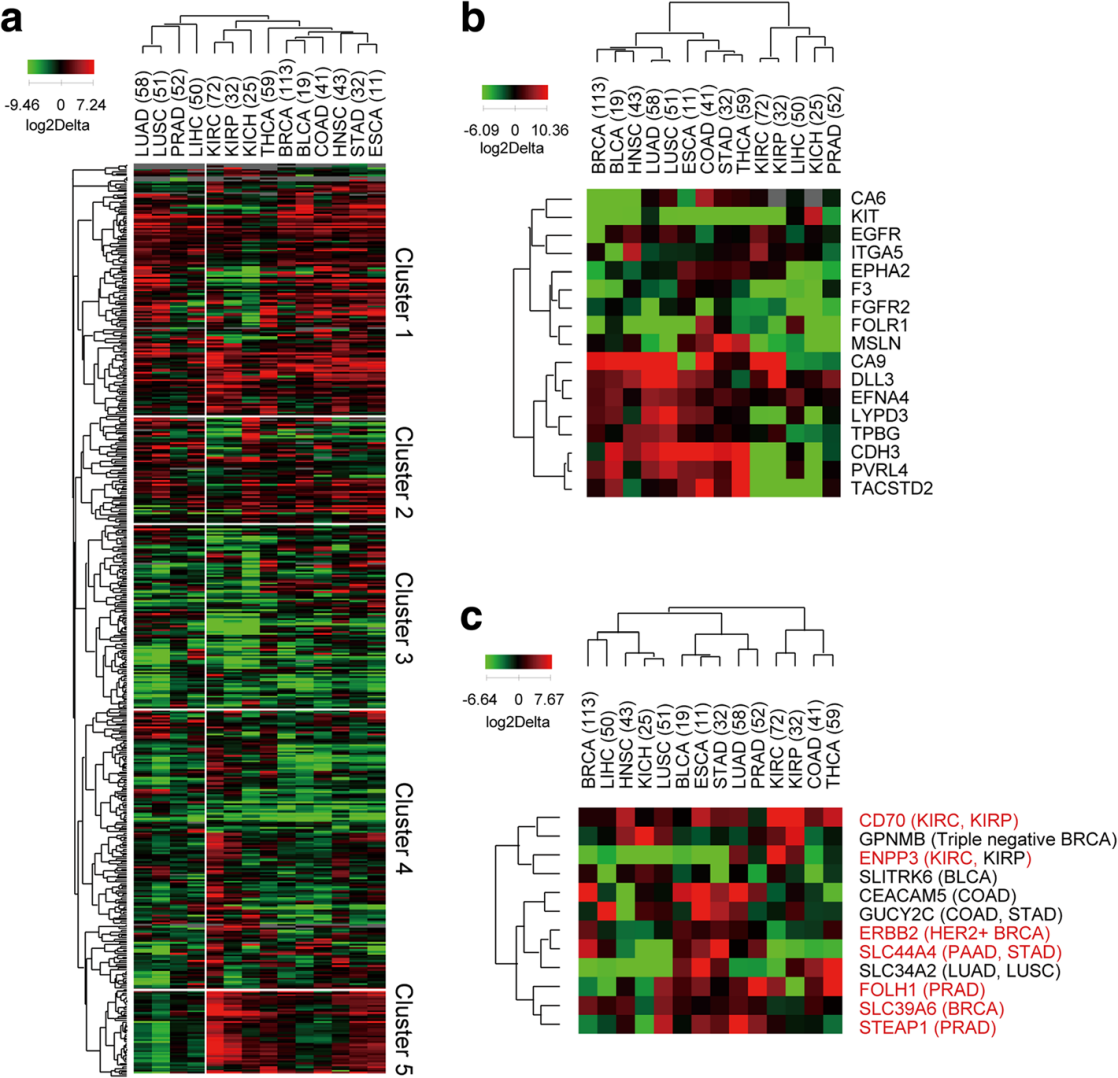
Hierarchical clustering of cell surface genes and known antigens for ADCs. **a** A heatmap of 519 cell surface genes and 14 cancer types. Heatmaps of ADC target genes differentially expressed on the diverse cancer types in (**b)** and other ADC target differentially expressed on the specific cancer types in (**c**). Cancer types are described in parentheness and significantly overexpressed target genes (log2Delta > 1 and *p* value < 0.01) in the specific cancer type are shown in red. QCanvas was used to cluster and draw heatmaps [32]

We analyzed the profile of 519 cell surface genes in 14 cancer lineages to show how many genes are over-expressed on diverse tumor samples or specific tumor samples via normal samples. The differentially expressed genes were classified into 5 groups (Fig. 3a). The genes Cluster 1 and 2 were over-expressed in multiple diverse lineages, while those in Cluster 5 were over-expressed in a subset of lineages and down-regulated in lung, prostate and liver cancer types. However, the genes belonging to Clusters 3 and 4 were relatively down-regulated in tumors compared to normal samples. Many of the genes in Cluster 4 were uniquely overexpressed in the kidney cancer type (KIRC). The lineage-wide distribution of all genes and 519 cell surface genes, and potential cell surface maker genes is shown in Additional file 1: Fig. S1.

Some of target antigens for ADCs are known to be expressed on tumor and normal tissue [23]. For example, target antigens over-expressed on specific cancer type, SLC34A2, translating NaPi2b, have high expressed in normal patients [24]. In case of RCC, membrane EGFR was expressed higher than tumor samples via normal samples. But the expression of cytoplasmic EGFR protein is lower than normal samples [25]. As described in Section 3.1, a total of 29 known target antigens of ADCs were analyzed in Fig. 3b and c. The 17 target antigens of ADCs which were known as over-expressed on diverse tumors, are actually classified into two groups (Fig. 3b). The upper cluster (CA6, KIT, EGFR, ITGA5, EPHA2, F3, FGFR2, FOLR1 and MSLN) exhibited lineage-dependent, limited expression patterns, while the bottom cluster (CA9, DDL3, EFNA4, LYPD3, TPBG, CDH3, PVRL4, and TACSTD2) is over-expressed on diverse tumors. The other 12 antigens of ADCs which were known as over-expressed on specific tumors, showed the over-expression on diverse lineages (Fig. 3c). Our analysis confirmed that seven genes (CD70, ENPP3, ERBB2, SLC44A4, FOLH1, SLC39A6 and STEAP1) were significantly over-expressed in the kn0own target lineages (in red), except ENPP3 in KIRP with log2Delta 1.06 and *P*-value 0.03.

### Identification of mutation-specific expression of surface genes

**Fig. 4:**
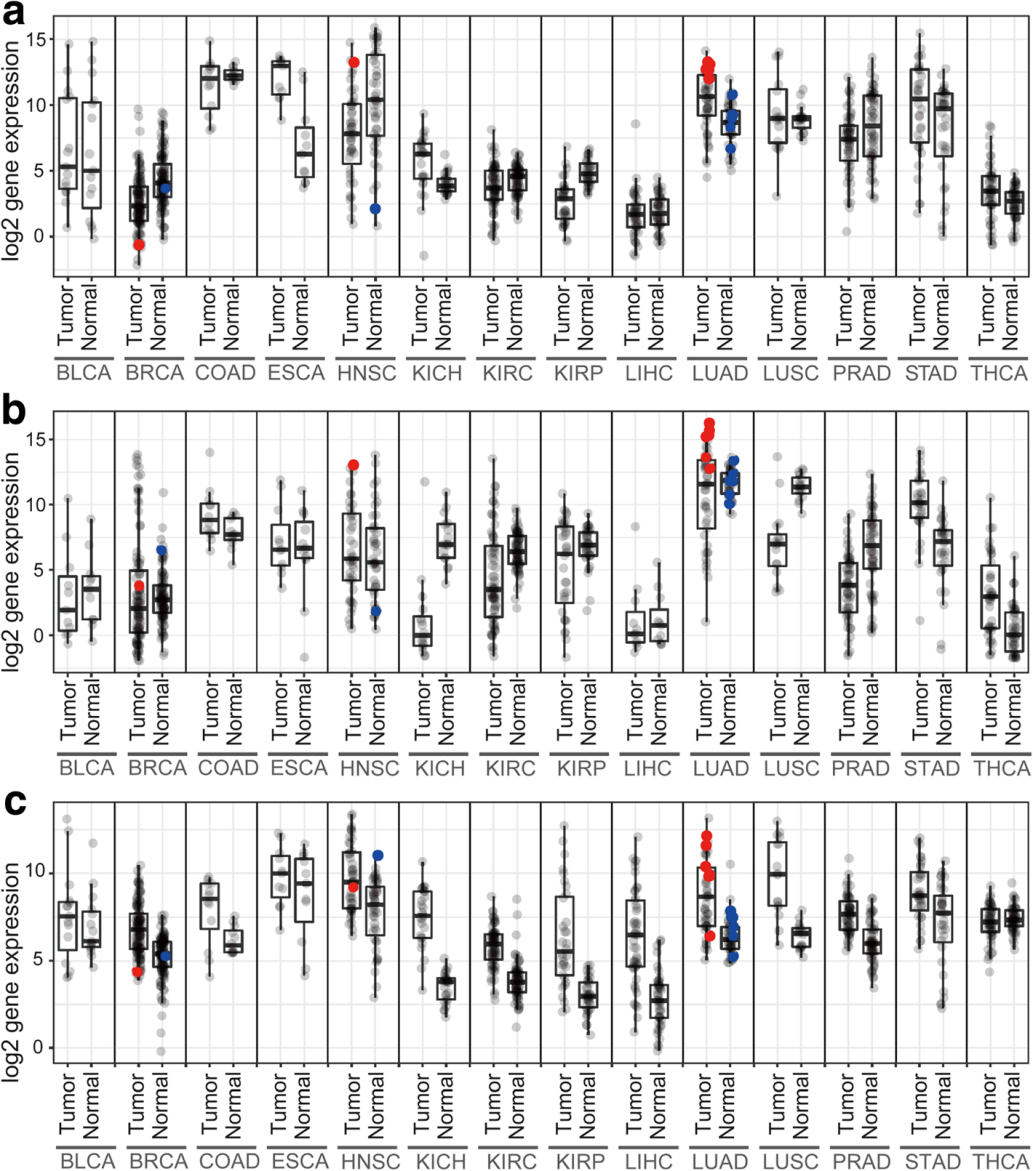
Comparison of MUC4 (**a**), MSLN (**b**) and SLC7A11 (**c**) expression profiles in 14 cancer types. Patient tumor samples with STK11 mutation and the corresponding normal samples are colored in red and blue, respectively. The number of patient samples with STK11 mutation BRCA = 1, HNSC = 1 and LUAD = 6

We analyzed the mutation-oriented profiles of gene expression in diverse lineages using QSurface. For example, the genes MUC4, MSLN, and SLC7A11 were predicted as STK11 mutation-specific cell surface markers in lung cancer samples (Fig. 4). Although MUC4 is not annotated using a ‘cell surface’ GO term, this transmembrane glycoprotein is differentially expressed on diverse cancer cell types, including LUAD [26, 27]. In the present study, MUC4 over-expression was observed in LUAD tumors compared to normal samples. Interestingly, this over-expression was highly associated with STK11 mutation in LUAD samples (log2Delta = 2.76, *P*-value = 0.002) (Fig. 4a). MSLN (or Mesothelin) is a known target gene over-expressed by the cells of solid tumors, particularly mesothelioma and LUAD [28]. MSLN-targeted ADC candidates are currently under investigation in phase I/II clinical trials for diverse cancer types. In the present analysis, we observed that MSLN expression was selectively associated with STK11 mutant samples (log2Delta = 4.74, P-value = 9.E-04) (Fig. 4b). Lastly, SLC7A11 is classified as a cell surface GO term, and this gene is known to be highly expressed by colon, kidney, and liver cancer cells [29]. Mutation-oriented analysis also revealed the strong association of SLC7A11 expression with STK11 mutations. The present mutant-oriented analysis of surface markers improved the statistical confidence in the selectivity of the expression of these genes in diverse cancer lineages. In the present study, MUC4, MSLN, and SLC7A11 showed high log2Delta values of 2.76, 4.74, and 1.55, with *P*-values of 0.002, 9.e-04, and 0.04, respectively.

### Validation of surface markers with advanced 3D assays

**Fig. 5:**
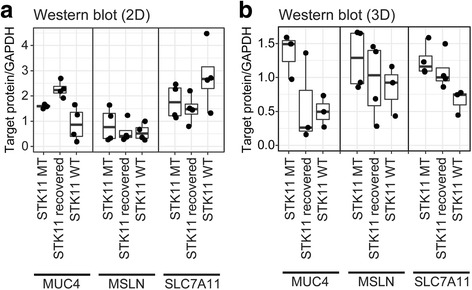
Comparison of MUC4, MSLN, SLC7A11 protein expression among STK11 mutant, recovered, and wild-type cell lines in 2D and 3D culture models. (**a**) *P*-values in 2D culture model are 0.04, 0.23, and 0.11 for MUC4, MSLN, and SLC7A11 resp. (**b**) P-values in 3D culture model are 0.01, 0.11, and 0.008 for the same order

Cancer cell culture system are classified into two-dimensional (2D) and three-dimensional (3D) cancer models [30]. Compared to a typical 2D monolayer cancer model, the 3D model mimics the in vivo environment because solid tumors grow in three-dimensions creating a unique microenvironment and facilitating cell-cell communication [31]. We measured the protein expression of the three surface markers (MUC4, MSLN and SLC7A11) in lung cell lines under 2D and 3D culture conditions. In the 2D system, the expression showed no difference between the STK11 mutant and wild-type cell lines of LUAD (Fig. 5a, Additional file 1: Fig. S2). However, the protein level of MUC4, MSLN, and SLC7A11 were significantly increased in STK11 mutant cell lines (Fig. 5b), confirming the transcription-level prediction of QSurface from patient samples. This result demonstrates the physiological relevance of the 3D sphere model for reproducing the expression feature of surface markers identified or predicted from patient samples. This validation confirms that QSurface provides useful and reliable tools for identifying mutation/lineage-specific surface markers and/or target antigens for ADCs.

## Conclusions

This study presents QSurface, rapid and efficient tools to identify novel tumor-specific cell surface markers for advanced cancer therapies. QSurface provides two analyzing method, lineage−/ and mutation-oriented profiles. To identify potential surface genes, QProfile used fold changes to find sensitivity of gene expression on given conditions. By using QSurface, we obtained 3 STK11-mutant specific expression markers, MUC4, MSLN, and SLC7A11 in LUAD. Furthermore, advanced 3D cell line models of lung cancer successfully reproduced the predict patterns by QSurface. And it demonstrates the physiological relevance of cell line-based 3D models with patient tumor data and confirms that QSurface is useful and reliable tools for identifying mutation/lienage-specific cell surface markers.

## Additional files

Additional file 1: **Table S1.** Data description of TCGA RNA sequencing data. **Table S2.** List of antibody-drug conjugates. **Figure S1.** Distribution of tumor sample-specific gene expression in 14 cancer types. Totally 20,531 genes, 519 cell surface marker, and significant cell surface hits (log2Delta > 1 and *p*-value < 0.01) are illustrated in grey, yellow, and red, respectively. **Figure S2.** Western blot analysis of the MUC 4, MSLN and SLC7A11 expression on STK11 mutant, restored and wild type cell lines. (A) gene expression on STK11 mutant, restored and wild-type cell lines on 2D culture status and (B) on 3D culture status. (DOCX 1902 kb)

## Abbreviations

2D: Two-Dimensional
3D: Three-Dimensional
ADC: Antibody-drug Conjugates
BRCA: Breast Invasive Carcinoma
CSLC: Cancer Stem-like Cell
GO: Gene Ontology
LUAD: Lung Adenocarcinoma
RSEM: RNA-Seq by Expectation Maximization
SLC: Stem-like Cell
TCGA: The Cancer Genome Atlas
