# Supplementary material for: QSurface: fast identification of surface expression markers in cancers
Source: BMC Syst Biol. 2018 Mar 19;12(Suppl 2):17. doi: 10.1186/s12918-018-0541-6 (PMC5861488; doi:10.1186/s12918-018-0541-6)
Supplement: Supplementary file 1 — Table S1. Data description of TCGA RNA sequencing data. Table S2. List of antibody-drug conjugates. Figure S1. Distribution of tumor sample-specific gene expression in 14 cancer types. Totally 20,531 genes, 519 cell surface marker, and significant cell surface hits (log2Delta > 1 and p-value < 0.01) are illustrated in grey, yellow, and red, respectively. Figure S2. Western blot analysis of the MUC 4, MSLN and SLC7A11 expression on STK11 mutant, restored and wild type cell lines. (A) gene expression on STK11 mutant, restored and wild-type cell lines on 2D culture status and (B) on 3D culture status. (DOCX 1902 kb) [file 12918_2018_541_MOESM1_ESM.docx]

**Supplementary Table 1.** Data description of TCGA RNA sequencing data.

| **Cancer**  **types** | **Full name** | **No. of paired**  **samples** | **No. of paired,**  **annotated**  **samples** | **No. of**  **annotated**  **mutations** |
| --- | --- | --- | --- | --- |
| BLCA | Bladder Urothelial Carcinoma | 19 | 15 | 1,500 |
| BRCA | Breast invasive carcinoma | 113 | 111 | 4,140 |
| COAD | Colon adenocarcinoma | 41 | 12 | 1,225 |
| ESCA | Esophageal carcinoma | 11 | 11 | 1,281 |
| HNSC | Head and Neck squamous cell carcinoma | 43 | 43 | 3,098 |
| KICH | Kidney Chromophobe | 25 | 25 | 1,336 |
| KIRC | Kidney renal clear cell carcinoma | 72 | 65 | 2,046 |
| KIRP | Kidney renal papillary cell carcinoma | 32 | 32 | 840 |
| LIHC | Liver hepatocellular carcinoma | 50 | 50 | 3,727 |
| LUAD | Lung adenocarcinoma | 58 | 45 | 3,698 |
| LUSC | Lung squamous cell carcinoma | 51 | 16 | 1,925 |
| PRAD | Prostate adenocarcinoma | 52 | 52 | 1,498 |
| STAD | Stomach adenocarcinoma | 32 | 32 | 5,487 |
| THCA | Thyroid carcinoma | 59 | 46 | 381 |
| **Total** |  | **658** | **555** | **13,646** |

**Supplementary Table 2.** List of antibody-drug conjugates.

| **Andibody-drug conjugates (ADC)** | **Target protein** | **Target gene** | **Target gene : full name** | **Indication** |
| --- | --- | --- | --- | --- |
| SGN-LIV1A | LIV1 | SLC39A6 | Solute Carrier Family 39 Member 6 | Breast |
| IMMU-130, SAR408701 | CEACAM5 | CEACAM5 | Carcinoembryonic Antigen Related Cell Adhesion Molecule 5 | Colorectal |
| MLN0264 | guanylyl cyclase | GUCY2C | Guanylate Cyclase 2C | Colorectal, Gastrointestinal |
| SYD985, Kadcyla, T-DM1, trastuzumab emtansine, PRO132365 | HER2 | ERBB2 | Erb-B2 Receptor Tyrosine Kinase 2 | Her2+ breast |
| DNIB0600A | NaPi2b | SLC34A2 | Solute Carrier Family 34 Member 2 | Ovarian, Lung |
| ASG-5ME | SLC44A4 | SLC44A4 | Solute Carrier Family 44 Member 4 | Pancreatic, Gastric |
| PSMA ADC, MLN2704 | PSMA | FOLH1 | Folate Hydrolase 1 | Prostate |
| DSTP3086S | STEAP1 | STEAP1 | STEAP Family Member 1 | Prostate |
| MDX-1203, BMS936561, AMG 172, SGN-CD70A | CD70 | CD70 | Tumor Necrosis Factor Ligand Superfamily Member 7 | Renal |
| AGS-16M8F, AGS-16C3F | ENPP3 | ENPP3 | Ectonucleotide Pyrophosphatase/Phosphodiesterase 3 | Renal, Prostate, Liver |
| PF-0626350 | 5T4 | TPBG | Trophoblast Glycoprotein | Solid tumors |
| SAR566658 | CA6 | CA6 | Carbonic Anhydrase 6 | Solid tumors |
| Bay 79-4620 | CA9 | CA9 | Carbonic Anhydrase 9 | Solid tumors |
| LOP628 | cKIT | KIT | proto-oncogene c-Kit | Solid tumors |
| SC16LD6.5, rovalpituzumab tesirine | DLL3 | DLL3 | Delta Like Canonical Notch Ligand 3 | Solid tumors |
| IMGN289 | EGFR | EGFR | Epidermal Growth Factor Receptor | Solid tumors |
| ABT-414 | EGFRvIII | EGFR | Epidermal Growth Factor Receptor | Solid tumors |
| MEDI-547, MI-CP177 | EphA2 | EPHA2 | ephrin type-A receptor 2 | Solid tumors |
| PF-06647263 | Ephrin-A4 | EFNA4 | Ephrin A4 | Solid tumors |
| BAY1187982 | FGFR2 | FGFR2 | Fibroblast Growth Factor Receptor 2 | Solid tumors |
| IMGN853 | FRα | FOLR1 | Folate receptor alpha | Solid tumors |
| BAY-94-9343 | mesothelin | MSLN | Mesothelin | Solid tumors |
| ASG-22CE | NECTIN4 | PVRL4 | Nectin Cell Adhesion Molecule 4 | Solid tumors |
| PCA062 | p-Cadherin | CDH3 | Cadherin-3 | Solid tumors |
| HuMax-TF-ADC | tissue factor | F3 | Coagulation Factor III, Tissue Factor | Solid tumors |
| IMMU-132 | TROP-2 | TACSTD2 | Tumor-Associated Calcium Signal Transducer 2 | Solid tumors |
| IMGN388 | αv integrin | ITGA5 | Integrin Subunit Alpha 5 | Solid tumors |
| BAY1129980 | C4.4a | LYPD3 | Ly6/PLAUR domain-containing protein 3 | Solid tumors |
| Glembatumumab vedotin | GPNMB | GPNMB | Glycoprotein Nmb | TN Breast |
| AGS15E | SLITRK6 | SLITRK6 | SLIT And NTRK Like Family Member 6 | Urothelial |


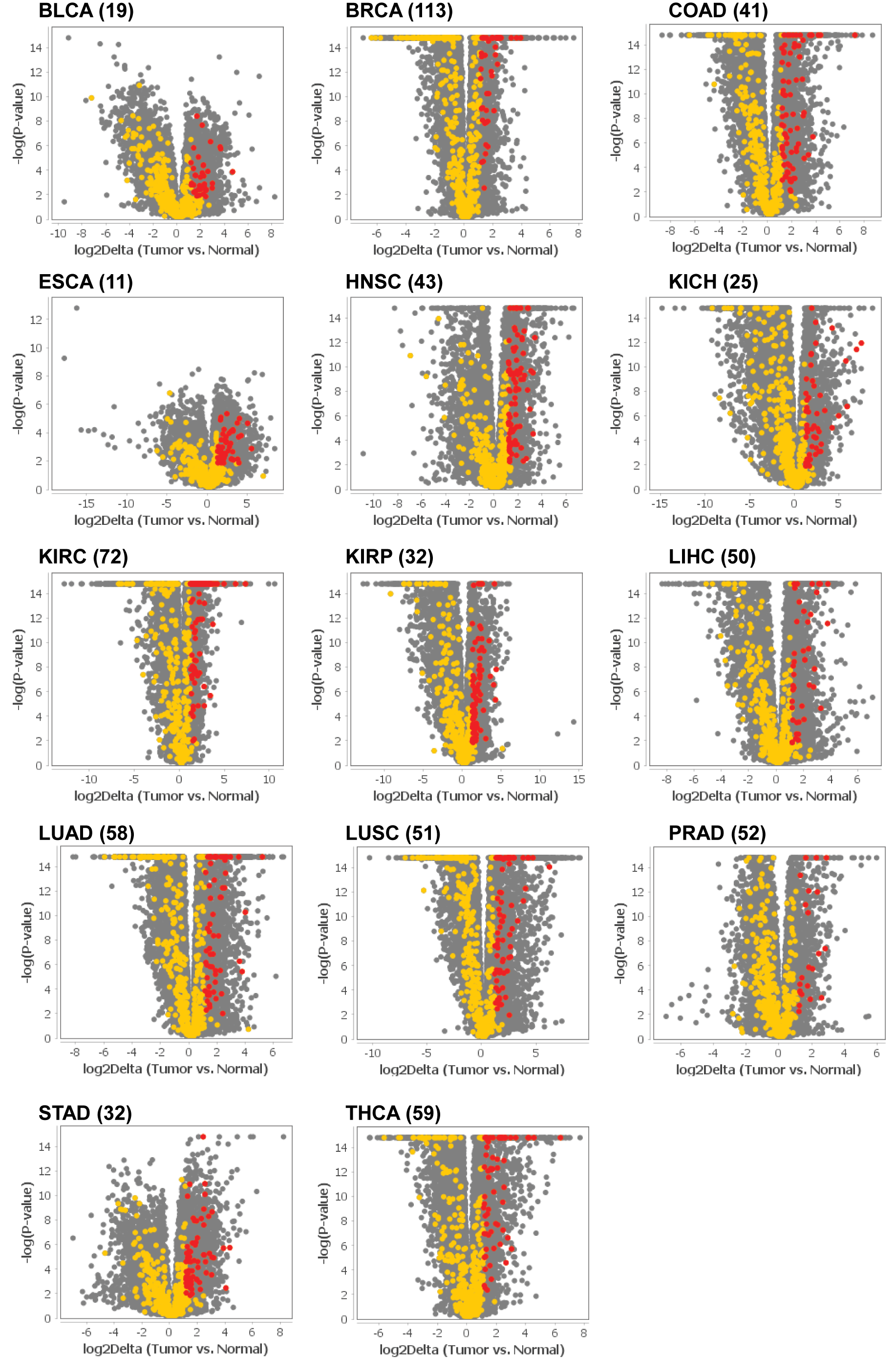


**Supplementary Figure 1.** Distribution of tumor sample-specific gene expression in 14 cancer types. Totally 20,531 genes, 519 cell surface marker, and significant cell surface hits (log2Delta > 1 and p-value < 0.01) are illustrated in grey, yellow, and red, respectively.


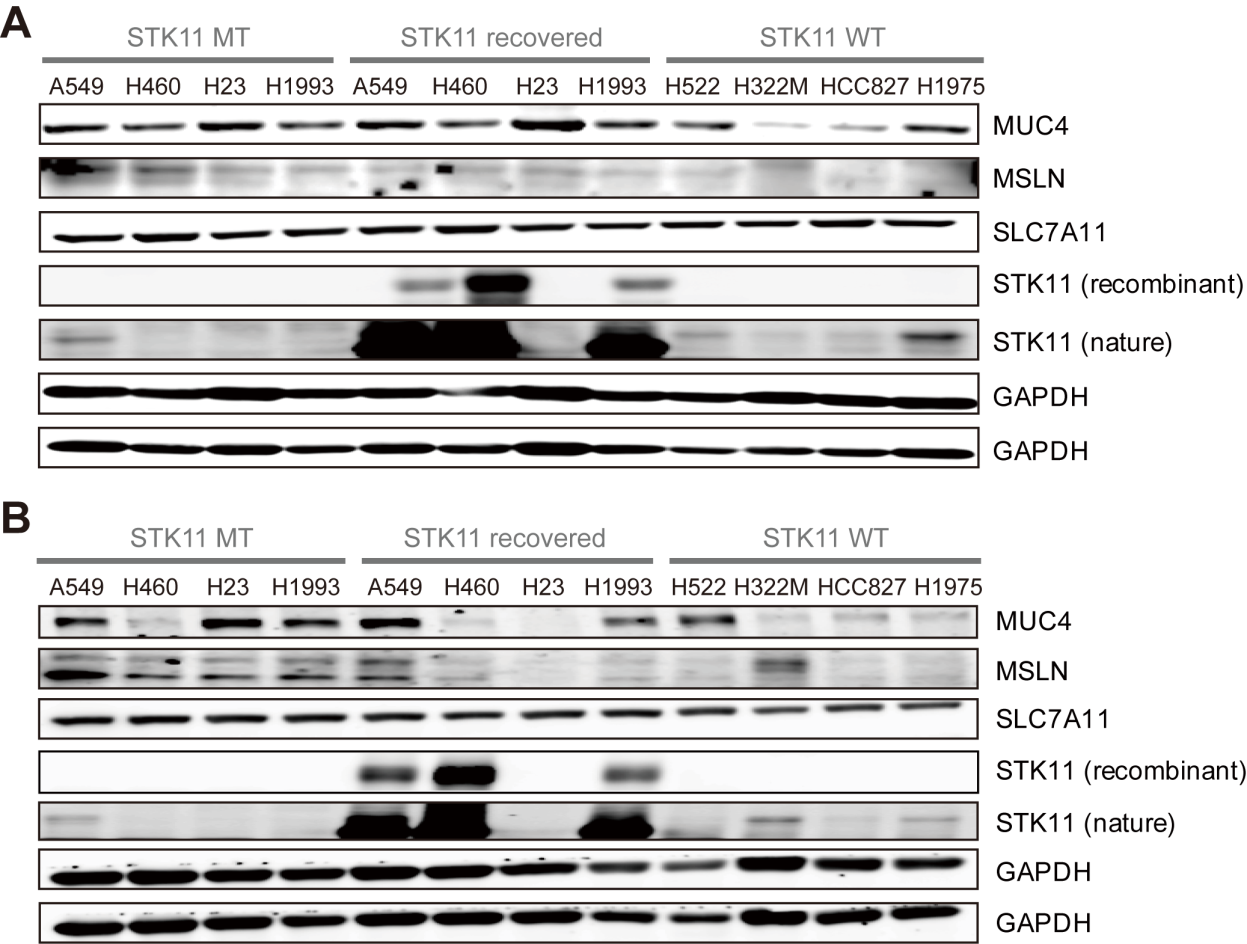
**Supplementary Figure 2.** Western blot analysis of the MUC 4, MSLN and SLC7A11 expression on STK11 mutant, restored and wild type cell lines. (A) gene expression on STK11 mutant, restored and wild-type cell lines on 2D culture status and (B) on 3D culture status.
